# A randomized, controlled, crossover study in patients with mild and moderate asthma undergoing treatment with traditional Chinese acupuncture

**DOI:** 10.6061/clinics/2015(10)01

**Published:** 2015-10

**Authors:** Hong Jin Pai, Raymundo Soares Azevedo, Alfésio Luís Ferreira Braga, Lourdes Conceição Martins, Beatriz M Saraiva-Romanholo, Milton de Arruda Martins, Chin An Lin

**Affiliations:** I Faculdade de Medicina da Universidade de São Paulo, Hospital das Clínicas, Instituto de Ortopedia e Traumatologia, Centro de Acupuntura, São Paulo, SP, Brazil; II Faculdade de Medicina da Universidade de São Paulo, Departamento de Medicina Interna, São Paulo, SP, Brazil; III Faculdade de Medicina da Universidade de São Paulo, Departamento de Patologia, São Paulo, SP, Brazil; IV Faculdade de Medicina da Universidade de São Paulo, Departamento de Patologia, Laboratório de Poluição Atmosférica Experimental, Grupo De Estudos de Epidemiologia Ambiental, São Paulo, SP, Brazil; V Universidade Católica de Santos, Programa de Pós-Graduação de Saúde Coletiva, Santos, SP, Brasil; VI Faculdade de Medicina da Universidade de São Paulo, Laboratório de Investigação Médica, LIM-20, São Paulo, SP, Brazil

**Keywords:** Asthma, Acupuncture, Sham, Spirometry, Quality of Life, Symptoms

## Abstract

**OBJECTIVES:**

This study sought to verify the effects of acupuncture as an adjuvant treatment for the control of asthma.

**METHODS:**

This was a randomized, controlled, crossover trial conducted at the Hospital das Clínicas da Faculdade de Medicina da Universidade de São Paulo. A total of 74 patients with mild/moderate, persistent asthma were randomized into two therapeutic groups: Group A – 31 patients underwent 10 real weekly acupuncture sessions, followed by a 3-week washout period and 10 sham weekly acupuncture sessions; and Group B - 43 patients underwent 10 sham weekly acupuncture sessions, followed by a 3-week washout period and 10 real weekly acupuncture sessions. Patients used short- and long-acting β-2 agonists and inhaled corticosteroids when necessary. Prior to treatment and after each period of 10 treatment sessions, the patients were evaluated for spirometry, induced sputum cell count, exhaled nitric oxide (NO) and with the Short Form 36 (SF-36) and Questionnaire on Quality of Life-Asthma (QQLA) questionnaires. Daily peak flow and symptom diaries were registered. The level of significance adopted was 5% (α=0.05).

**RESULTS:**

In Group B, after real acupuncture, there was a decrease in eosinophils (*p*=0.035) and neutrophils (*p*=0.047), an increase in macrophages (*p*=0.001) and an improvement in peak flow (*p*=0.01). After sham acupuncture treatment, patients experienced less coughing (*p*=0.037), wheezing (*p*=0.013) and dyspnea (*p*=0.014); similarly, after real acupuncture, patients reported less coughing (*p*=0.040), wheezing (*p*=0.012), dyspnea (*p*<0.001) and nocturnal awakening episodes (*p*=0.009). In Group A, there was less use of rescue medication (*p*=0.043). After the sham procedure, patients in Group A experienced less coughing (*p*=0.007), wheezing (*p*=0.037), dyspnea (*p*<0.001) and use of rescue medication (*p*<0.001) and after real acupuncture, these patients showed improvements in functional capacity (*p*=0.004), physical aspects (*p*=0.002), general health status (*p*<0.001) and vitality (*p*=0.019). Sham acupuncture also led to significant differences in symptoms, but these were not different from those seen with real acupuncture. Spirometry and exhaled NO levels did not show a difference between sham and real acupuncture treatment. In addition, no significant difference was demonstrated between treatments regarding the quality of life evaluation.

**CONCLUSION:**

Real and sham acupuncture have different effects and outcomes on asthma control. The crossover approach was not effective in this study because both interventions led to improvement of asthma symptoms, quality of life and inflammatory cell counts. Thus, sham acupuncture cannot serve as a placebo in trials with acupuncture as the main intervention for asthma.

## INTRODUCTION

Asthma is a chronic condition with a global incidence that has progressively increased in recent decades 1. It is estimated that approximately 300 million people are currently affected by asthma 1. Asthma is defined as a condition that causes chronic inflammation in the airways, leading to a state of hyper-responsiveness and resulting in bronchospasm, an increase in mucus production and limitation to air flow 1. The most common symptoms of asthma are wheezing, coughing, chest tightness and dyspnea. Pharmacological treatment includes short-acting and some long-acting β-2 agonists, glucocorticoids (both oral and inhaled), a leukotriene modifier and theophylline, among others 1. Although medications and updated approaches have been effective tools for controlling asthma, many other approaches have been studied to help asthmatic patients reach both symptom control and improvement in their quality of life, particularly non-conventional healing techniques 2-4. Among these non-conventional approaches, termed alternative/complementary medicine, acupuncture has become one of the most popular techniques used to control asthma 5.

Acupuncture is one of several traditional Chinese medicine therapeutic methods. For thousands of years, acupuncture has been used to treat several conditions, including asthma 6. In comparison to mainstream medicine, acupuncture is considered a complementary/alternative healing technique and has been considered to be effective for conditions such as nausea and emesis post-chemotherapy, dental pain, osteoarthritis pain and headache, among others 7. The apparent lack of significant adverse effects, unlike those experienced with the use of glucocorticoids and the possible effects linked to the release of adrenocorticotropic hormone (ACTH) and endogenous corticoids, supports the use of acupuncture for the treatment of asthma. Several studies have been conducted to investigate acupuncture in the treatment of asthma, with controversial results 6,. Most previous studies favoring acupuncture for the treatment of asthma have been published in non-English language journals and they lack good epidemiological design 10. Indeed, there are some inherent difficulties in designing and establishing placebo controls in studies involving acupuncture 11,12. Due to the nature of heterodox methods of diagnosis, some diseases or conditions can be understood as having different etiologic agents, diverse pathophysiologic mechanisms and different methods of treatment. Thus, more studies about the effect of acupuncture in asthma are needed.

To better study and perhaps understand the possible effects of acupuncture in mild and moderate asthma, we designed and performed the present study in the Clinics Hospital of the University of São Paulo School of Medicine, one of the largest medical centers in Latin America, using the following primary end-points: symptomatic and quality of life improvement, spirometry and peak flow improvement and inflammatory improvement (cell counting and nitric oxide (NO) measurements).

### Strengths and limitations of this study

This study attempted to evaluate the effects of acupuncture on patients with asthma in a controlled, randomized, double-blind crossover trial and it may have shown the positive effects of acupuncture for the following end-points:

1. Symptom control
2. Cell counts in induced sputum
3. Peak flow
4. Quality of life

However, there are several limitations of the study:

1. A significant number of patients quit the study prior to completion
2. No significant difference was demonstrated between the sham and real acupuncture treatments regarding the quality of life evaluation

## MATERIAL AND METHODS

This was a double-blind (patient and clinicians), randomized, crossover clinical trial study where all enrolled patients underwent both sham and real acupuncture. A specific calculation for sample size was not performed because there was no defined effect to reach with the qualitative variables (quality of life evaluation, symptoms, etc.). We recruited patients through the written press and performed the first interview by phone to evaluate the general conditions needed for enrollment in the study. After the first interview, the patients were taught how to answer a daily symptom scale, perform peak flow measurements and answer quality of life questionnaires, including the Short Form 36 (SF-36) and Questionnaire on Quality of Life-Asthma (QQLA). Afterwards, a second interview was conducted and the volunteers were invited by research staff to participate in the project.

The quality of life questionnaires were completed initially, before beginning the treatment procedure and also after each treatment period (sham and real acupuncture). Hence, each patient answered both the questionnaires three times. These questionnaires are useful instruments for measuring quality of life in respiratory-impaired patients. Some of the questions were particularly useful, such as those regarding physical limitations, functional capacity and the frequency and severity of symptoms.

A total 184 patients were included according to the following criteria: non-smokers; forced expiratory volume of first second (FEV_1_) ≥ 70% and peak flow variability ≤ 20%; asthma symptoms at least twice per week and a maximum 5 times per week; nocturnal awakening < 4 times per month; use of relieving inhaled β-2 rapid-acting/long-acting or inhaled glucocorticoids; and lack of other cardiovascular disease or chronic obstructive pulmonary disease (COPD).

Patients interested in participating but not enrolled to this study were excluded according to the following criteria: participating in another clinical trial; co-morbidity with other lung or systemic disease; past bleeding or coagulopathy; recent asthma exacerbation by upper airway infection; use of antidepressant medication; pregnancy; smoker or ex-smoker who quit less than 1 year ago; or drug addict.

The enrolled patients were allocated randomly, using a simple random sampling method, to 2 groups and had their spirometry, sputum cell count, NO measurement and medical interview performed before they began the treatment. During this period of inclusion, the patients were taught how to perform daily peak flow measurements and how to complete daily symptom scales. Subsequently, they were randomly allocated into one of two treatment groups: 1) Group A, in which patients first received real acupuncture for 10 weekly sessions and 2) Group B, in which patients first received sham acupuncture treatment with points not related to the treatment of asthma in traditional Chinese medicine (some are not described as acupoints) for 10 weekly sessions.

After 10 sessions, the patients in both groups underwent spirometry and induced sputum cells counts and NO measurements were again performed. Both groups underwent a 3-week washout period and then performed the crossover process, in which Group A and Group B exchanged the form of needling, with Group A receiving sham point needling and Group B receiving real acupoint needling. After 10 weekly sessions, new objective measurements were performed. During each study period, patients suffering an asthma exacerbation were rescued with β-agonists (both short-and long-acting) and inhaled glucocorticoids. During the period of treatment, 36 patients used short-acting β-2 agonists as rescue medications, 6 patients used inhaled corticosteroids, 49 patients used a combination of short-acting β-2 and long-acting β-2 agonists with inhaled corticosteroids, 3 patients used β-2 short-acting agonists with inhaled corticosteroids and a leukotriene modifier and 2 used theophylline and a short-acting β-2 agonist. The procedure is summarized in Figure 1.

The patients were randomly allocated; there was only one physician who performed acupuncture on the patients. Neither the patient, nor the interviewer, nor the technician who measured the induced sputum cell counts, NO measurements and spirometry had knowledge of the type of acupuncture (real or sham) the patients were undergoing. In the end, a total of 74 patients achieved a total of 20 sessions of treatment, comprising 10 with real acupuncture and 10 with sham acupuncture.

All of the patients underwent their laboratory exams in the Clinics Hospital of the University of São Paulo Faculty of Medicine and underwent the sessions of treatment in a location specifically prepared to perform this study.

The local Committee for Ethical Issues in Human Research approved this study.

### Statistical analysis

The quantitative continuous variables were analyzed using the student's t test for two independent samples or by paired t test, as appropriate.

The ordinal qualitative variables were analyzed using the Mann-Whitney test for two independent samples or by Wilcoxon test for two paired samples.

The nominal qualitative variables were analyzed using the Chi-Squared test or Fisher Exact test, as appropriate.

We adopted a statistical significance level of 5% (α=0.05). The analyses were performed using Minitab 15.0 and SPSS 16.0 for Windows.

## RESULTS

From January 2003 to December 2007, a total 185 volunteers were recruited. Of these, only 74 completed the study by passing through both the sham and real acupuncture treatment groups. Forty-three volunteers began with the sham technique (Group B), passed through the washout period and subsequently received real acupuncture treatment. Thirty-one subjects were allocated to the group that first received real acupuncture (Group A) and after a washout period, the subjects passed to the sham acupuncture treatment.

The groups of volunteers comprised 52 females (70%) and 22 males (30%), with a mean age of 32.5 years for Group A (acupuncture as the first procedure) and 37.4 years for Group B (sham as the first procedure). Because this was a very long-term trial, we had a considerable loss of patients. A total 111 volunteers quit the trial for several reasons such as job overload, moving from former addresses, asthma symptoms brought under control during the trial and other concurrent diseases occurring while undergoing the trial. We also had problems with volunteers not filling out the proposed questionnaires, making it impossible to use these questionnaires for evaluation.

No patients reported any harm or adverse effects during the period of intervention.

### Peak flow

There was a significant increase in peak flow measurements in Group B patients after the real acupuncture intervention (Table 1).

### Cell counts in induced sputum analysis

There was a significant decrease in the eosinophil and neutrophil counts and a significant increase in the macrophage count in the induced sputum in Group B subjects after the real acupuncture procedure (Table 2).

### Self-report of daily symptoms scale

In terms of daily symptoms related to asthma, there was a significant decrease among Group A subjects regarding the β-2 agonists required, comparing the inclusion period to the period after the real acupuncture procedure. In addition, there was a significant decrease in coughing, wheezing, dyspnea and β-2 agonist spray use, comparing the inclusion period to that after the sham procedure (Table 3).

In Group B, there was also a significant decrease in coughing, wheezing, and dyspnea, comparing the inclusion period to that after the sham procedure. Comparing the inclusion period to that following real acupuncture treatment, there was also a significant decrease in coughing, wheezing, dyspnea and nocturnal awakening (Table 3).

### Quality of life evaluation

#### A- Questionnaire on Quality of Life-Asthma, Universidade Federal de São Paulo, Escola de Medicina (QQLA)

The results showed improvement in some domains of the QQLA and this improvement was observed after both the real acupuncture and after the sham procedure. However, after the real acupuncture, more domains showed improvement than after the sham procedure. In this questionnaire, patients experienced improvement in domains such as physical limitation, socio-economic aspects, symptom frequency and severity, adherence to treatment and social and psychological aspects (Table 4). However, there was no significant difference between the real acupuncture and the sham procedure treatments.

### SF-36 medical outcome study

As described above for the QQLA, we found a very similar result for the SF-36. Both the sham and real acupuncture treatments seemed to improve domains related to functional capacity, physical aspects, general health status, mental health, social aspects and vitality (Table 5). However, there was no difference in this improvement when comparing sham and real acupuncture.

We also found no difference comparing the inclusion period to both the sham and real acupuncture periods for other measurements such as spirometry and NO exhaled measurements.

## DISCUSSION

For thousands of years, acupuncture has been used as a first-choice treatment for several illnesses on the Asian continent, mostly in countries such as China, Japan and Korea, as well as in Southeast Asia 6. Although the efficacy of acupuncture remains unknown (because of a lack of reliable case records from the distant past), most traditional Chinese medicine practitioners claim that acupuncture is effective in asthma symptom control 10.

This study was conducted to test the consistency of the positive effect of acupuncture in asthma control. We decided not only to study symptom control but also to determine whether acupuncture has some influence on small airway inflammation; thus, we collected data on spirometry, total cell counts in induced sputum and NO measurements. We also collected data on daily symptom scales and measured quality of life through two pertinent questionnaires.

The design of our study was a crossover type, to facilitate recruitment by not having to recruit separate control, sham (placebo) and real acupuncture groups (treatment group). The aim was that during the enrolling period, once we had collected the measurements for spirometry, the symptom scale, quality of life questionnaire, total cell counts in induced sputum and NO, the patients would be considered a control group. Afterwards, while each one of the patients passed throughout the different stages of treatment, they would be delegated to a placebo (sham) group or a treatment (acupuncture) group. This type of design was quite risky because we could not find another similar study to establish an acceptable period of washout for the acupuncture effect. We decided to extend the well-validated washout period of two weeks for corticosteroids to a period of three weeks, without any certainty if the duration of this period would be long enough. The lack of difference between the sham and real acupuncture found at the end of this study may be accounted for by ineffectiveness of the washout period.

Due to the duration of the study period, which lasted longer than expected (more than 4 years), we experienced a considerable loss of volunteers who quit the study for several reasons, as listed in Table 6. Although alarming, this loss is expected in clinical trials lasting for long periods of time 16,17. This loss of subjects is possibly the main weakness of our study and prevented us from drawing convincing conclusions about the contribution of acupuncture to asthma treatment.

As this study took a long time to be completed (more than 4 years), we could not perform good seasonality control, given periods of asthma exacerbation, with use of inhaled glucocorticoids and both long- and short-acting β-agonists. Although a period of washout was established every time rescue medications were required, we could not be certain about distinguishing both the acupuncture (either real or sham) and rescue medication effects for the patients; this fact certainly impaired the strength of our study.

Thus, the results showing no difference in NO measurements and spirometry could be accounted for by poor seasonality control, rescue medication use and an ineffective washout period. Additionally, due to the heavy use of rescue medications, including inhaled glucocorticoids, we expected that the inflammatory process (expressed in NO measurements) would show differences between the pre-treatment group and both the acupuncture and sham groups, although this was not observed. This finding led us to think that perhaps most asthma patients with controlled symptoms have silent, chronic airway inflammation. In contrast, we observed a decrease in eosinophils and neutrophils, especially in Group B, after the second intervention (real acupuncture), which may point to the inflammation-reducing effects of acupuncture. Moreover, there was an increase in the macrophage count after real acupuncture in Group B, suggesting a positive modulatory effect of real acupuncture on increasing the macrophage count, which is important in the atopic allergy process 16.

Other results of note include the improvement of peak flow in Group B after the real acupuncture intervention; this fact again points to the possible inflammation-reducing effect of acupuncture.

We had difficulty establishing the sham points as a “true placebo”. Classically, sham points are defined as distant points and may or may not technically have any therapeutic effects when stimulated 12,17. However, there remains no consensus about establishing sham points and there are studies suggesting that sham points could have some minimal therapeutic effects because any aching point on the skin could serve as an acupuncture point 12,17. Therefore, we could not distinguish significant effects between treatment (acupuncture points) and placebo (sham points), particularly in the symptoms and quality of life evaluation, which led us to conclude that sham acupuncture may have therapeutic, yet minimal effects. We selected many acupoints in distant meridians that could have an influence on asthma control, but we could not assure that these points really had no effect on asthma. This fact could have contributed to the lack of difference in the endpoints between the real and sham acupuncture in the subjective fields of measurements (symptoms and quality of life). This limitation could also explain the difference in effectiveness between the real acupuncture points and the sham points during nocturnal awakening, the decrease in use of rescue medication and the quality of life questionnaire. Thus, our results support the concept that sham acupuncture is not possible, only acupuncture delivering minimal effects 16.

The results of our study appear to suggest that acupuncture does play an effective role in airway inflammation because positive results were found in peak flow measurements and decreases in the eosinophil and neutrophil counts in the induced sputum and the increase in macrophages. These findings showed a positive modulating effect on the immune system, although no effects on spirometry or exhaled NO measurement were found. In contrast, in terms of symptoms, acupuncture (together with known medications) may offer better symptom control and a better quality of life.

However, the number of volunteers who quit this study may have compromised the measurement of the real effect of acupuncture, prohibiting us from making a more positive assumption about the effect of acupuncture. The lack of a significant difference between the sham and real acupuncture treatments also prevented us from deducing the effectiveness of real acupuncture. However, sham acupuncture may not be considered an effective placebo as it showed an effect in this study.

Based on the points discussed above, we believe that the crossover study design does not appear to be appropriate for the evaluation of the effect of acupuncture, particularly because we did not establish a reliable washout period and sham acupuncture showed effects on asthma control.

Further studies are required to better understand the effects of acupuncture in asthma, perhaps using other type of devices - such as a needle that does not penetrate the skin but gives the sensation of being pricked - to better fit the condition of sham acupuncture points. In addition, the crossover design may not be appropriate for the evaluation of acupuncture effects.

Acupuncture, together with other medications (like β-agonists, regardless of whether they are short- or long-acting, leukotriene modifiers or inhaled glucocorticoids), may have a positive effect on symptom control and improvements in quality of life, in addition to possible effects on the control of chronic airway inflammation. However, due to several reasons, including a significant number of volunteers who quit before the end of the study protocol, we cannot guarantee these effects of acupuncture on asthma.

## Figures and Tables

**Figure:**
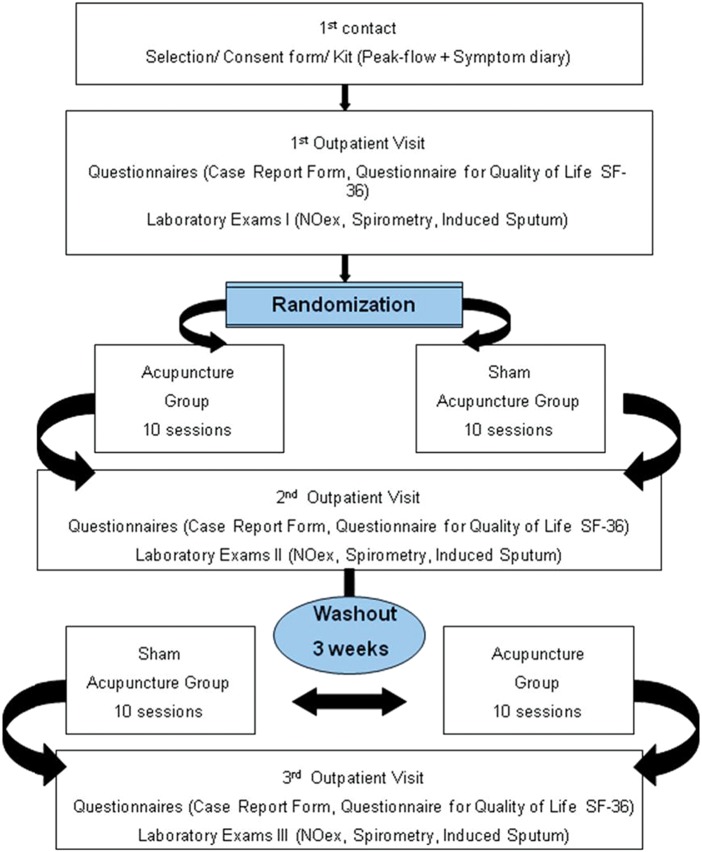
Protocol fluxogram showing how patients enrolled in the study were divided and treated.

**Table 1:** Peak flow measurements compared with baseline after real acupuncture in Group B.

	Peak Flow (L/minute)		
	Mean	Standard Deviation	Standard Error of Mean
Before any intervention	324.3	95.0	15.6
Real Acupuncture	345.8	92.4	15.2
Differences	−21.51	48.42	7.96

*p*=0.010

**Table 2:** Difference in total eosinophil, neutrophil and macrophage counts in Group B between the inclusion period and after the second intervention (real acupuncture).

White Cells	N	Mean	Standard Deviation	Standard Error of Mean
Eosinophils				
Period of inclusion	46	10.97	20.29	2.99
After real acupuncture	46	5.21	13.16	1.94
Difference	46	5.76^1^	18.01	2.66
Neutrophils				
Period of inclusion	46	49.26	28.05	4.14
After real acupuncture	46	38.71	22.91	3.38
Difference	46	10.55^2^	34.96	5.15
Macrophages				
Period of inclusion	46	37.39	25.94	3.82
After real acupuncture	46	54.04	25.93	3.82
Difference	46	−16.65^3^	31.17	4.60

1 - 95% CI for mean difference: (0.41; 11.11); *p*=0.035

2 - 95% CI for mean difference: (0.17; 20.93); *p*=0.047

3 - 95% CI for mean difference: (-25.91; -7.39); *p*=0.001

**Table 3:** Daily self-report symptom scale in both Groups A and B after real acupuncture or sham interventions compared to the inclusion period parameters.

	GROUPS AND EXPOSURES					
	Group A - after real acupuncture			Group A - after sham acupuncture		
Symptoms	Median	Wilcoxon Test	*p*	Median	Wilcoxon Test	*p*
Coughing	−0.05000	89.0	0.140	−0.1250	61.0	0.007
Wheezing	−0.07250	129.5	0.096	−0.09000	101.5	0.037
Dyspnea	−0.1100	127.0	0.052	−0.1925	45.0	0.000
Nocturnal awakening	−0.01500	118.5	0.376	−0.05500	90.5	0.092
ß-2 Agonist spray use	−0.07000	113.5	0.043	−0.1950	4.5	0.000

	Group B - after sham acupuncture			Group B - after real acupuncture		
	Median	Wilcoxon Test	*p*	Median	Wilcoxon Test	*p*
Coughing	−0.04500	200.0	0.037*	−0.06000	189.0	0.040*
Wheezing	−0.09000	186.5	0.013*	−0.1150	185.0	0.012*
Dyspnea	−0.08000	254.5	0.014*	−0.1500	126.5	0.000*
Nocturnal awakening	−0.01500	244.5	0.369	−0.04500	123.0	0.009*
ß-2 Agonist spray use	0.000000000	320.0	0.844	−0.01500	246.0	0.383

**Table 4:** Evaluation of the Questionnaire of Quality of Life in Asthma (QQLA) domains before (inclusion period) and after both the real and sham interventions.

	GROUPS AND EXPOSURES					
	Group A - after real acupuncture			Group A - after sham acupuncture		
Domains in QQLA-UNIFESP	Median	Wilcoxon Test	*p*	Median	Wilcoxon Test	*p*
Physical limitation	−0.0833	74.0	0.006*	−0.0450	144.5	0.117
Symptom frequency and severity	−0.0898	71.0	0.001*	−0.0833	74.5	0.001*
Adherence to treatment	−0.0417	71.0	0.043*	−0.0416	68.0	0.173
Socio-economic aspects	−0.0208	80.0	0.080	0.0000	61.5	0.306
Socio-professional aspects	−0.0179	75.5	0.059	0.0000	124.0	0.681
Psychosocial aspects	−0.0156	95.0	0.314	0.0000	164.5	0.968
Social aspects	−0.0518	56.5	0.00*	−0.0406	110.5	0.021*

	Group B - after sham acupuncture			Group B - after real acupuncture		
	Median	Wilcoxon Test	*p*	Median	Wilcoxon Test	*p*
Physical limitation	−0.0333	284.0	0.212	−0.1167	181.5	0.001*
Symptom frequency and severity	−0.0448	331.5	0.089	−0.0449	266.5	0.086
Adherence to treatment	0.0000	268.0	0.620	0.0000	366.0	0.833
Socio-economic aspects	−0.0416	156.5	0.010*	−0.0624	141.5	0.002*
Socio-professional aspects	−0.0000	242.5	0.503	−0.0357	67.5	0.001*
Psychosocial aspects	−0.0156	127.5	0.032*	−0.0234	150.5	0.057
Social aspects	−0.0264	277.0	0.018*	−0.0508	231.0	0.004*

**<?ENTCHAR ast?>:** *p*<0.05

**Table 5:** Evaluation of the SF-36 domains before (inclusion period) and after both the real and sham interventions.

	GROUPS AND EXPOSURES					
	Group A - after real acupuncture			Group A - after sham acupuncture		
Domains in SF-36	Median	Wilcoxon Test	*p*	Median	Wilcoxon Test	*p*
Functional capacity	7.50	252.0	0.004*	7.50	252.5	0.016*
Physical aspects	25.00	187.5	0.002*	25.00	199.0	0.004*
Pain	5.00	163.0	0.242	0.00	163.5	0.447
General health status	7.50	224.0	0.000*	7.50	259.0	0.010*
Vitality	7.50	268.5	0.019*	10.00	283.0	0.025*
Social aspects	6.25	169.5	0.168	6.25	235.5	0.131
Emotional aspects	0.00	82.0	0.485	0.00	90.5	0.255
Mental health	2.00	220.0	0.464	4.000	243.0	0.368

	Group B - after sham acupuncture			Group B - after real acupuncture		
	Median	Wilcoxon Test	*p*	Median	Wilcoxon Test	*p*
Functional capacity	7.50	501.5	0.002*	10.00	625.5	0.001*
Physical aspects	12.50	265.5	0.023*	25.00	344.0	0.001*
Pain	0.00	270.5	0.666	5.00	324.5	0.021*
General health status	5.00	441.0	0.014*	10.00	570.5	0.000*
Vitality	5.00	473.5	0.028	10.00	543.5	0.001
Social aspects	6.25	351.0	0.211	12.50	389.5	0.006
Emotional aspects	0.00	263.0	0.175	16.67	284.0	0.067
Mental health	−2.00	313.5	0.765	4.00	506.5	0.049*

**<?ENTCHAR ast?>:** *p*<0.05

**Table 6:** Descriptive data on the number and reasons for patients quitting the trial.

Reason for Quitting	N	%
Work overload	30	27.5
Other diseases	14	12.8
Moved to other location	6	5.5
Fear of needling/shyness	4	3.6
No symptoms	1	0.9
Family problems	1	0.9
No reason	38	32.0
Not answering the questionnaires	14	12.8
Robbed while undergoing study	1	0.9
Total	109	100.0
